# Own Pain Distress Mediates the Link Between the Lifestyle Facet of Psychopathy and Estimates of Pain Distress in Others

**DOI:** 10.3389/fnbeh.2022.824697

**Published:** 2022-02-24

**Authors:** Inti A. Brazil, Dimana V. Atanassova, Joukje M. Oosterman

**Affiliations:** ^1^Donders Institute for Brain, Cognition and Behavior, Radboud University, Nijmegen, Netherlands; ^2^Forensic Psychiatric Centre Pompestichting, Nijmegen, Netherlands

**Keywords:** psychopathy, pain, distress, moral decision-making, social cognition, pain distress

## Abstract

Psychopathy is a multifaceted personality construct entailing interpersonal-affective disturbances, antisocial traits, and a tendency to lead an erratic lifestyle. Elevated levels of psychopathic traits have been linked to having an altered experience of pain, reduced responsivity to distress in others, and making poor moral choices that bring harm to others. In the context of moral decision-making, it is possible that the capacity to estimate the distress felt by others is linked to a limitation in the first-hand experience of distress, as the presence of psychopathic traits increases. We employed a model-based approach in a non-offender sample (*n* = 174) to investigate whether pain-related distress mediated the links between facets of psychopathy and estimates of the pain distress potentially experienced by others. Participants judged the permissibility of moral dilemmas and rated how much pain distress they would experience while making such judgements, as well as how much pain distress they believed the “victims” would feel as a result of the moral choice made by the participant. We found that ratings of own pain distress predicted beliefs about the distress others may experience, and elevated scores on the lifestyle facet of psychopathy uniquely predicted lower estimates of own pain distress. Furthermore, own pain distress mediated the relationship between the lifestyle facet and beliefs about others’ distress. Finally, exploratory zero-order correlation analyses revealed that ratings of own pain distress decreased as the scores on multiple psychopathic traits increased. Only the lifestyle facet correlated in the negative direction with beliefs about others’ distress. Taken together, our findings suggest that beliefs about how much pain distress others may experience is indeed mediated by own pain distress, and that the tendency to lead an erratic lifestyle is linked to alterations in this mechanism.

## Introduction

Psychopathy is a personality construct typified by disrupted interpersonal and affective functioning (e.g., lack of guilt, reduced empathy, manipulativeness), combined with a tendency to have an erratic lifestyle and to engage in disruptive and antisocial behavior (Hare, 2003). Although psychopathy has often been studied as a categorical construct in clinical settings, a growing amount of evidence points to the dimensional nature of the behavioral features of psychopathy, with severe cases scoring in the higher end of a continuum (Neumann et al., 2012). One advantage of the dimensional approach to psychopathy is that it makes it possible to measure the corresponding traits in the general community as well, which can help identify the impaired mechanisms that are associated with particular psychopathic traits. Research in both offender and community samples has consistently pointed out that individuals with elevated levels of psychopathic traits often inflict harm on others, in part due to lack of moral constraints (Blair, 1995, 2007). Persistent engagement in aggressive, often exploitative behavior with no apparent concern for the victim, is currently seen as a hallmark of having elevated levels of psychopathic traits (Brislin et al., 2016).

In most individuals, the prospect of inflicting harm is enough to prevent behaviors resulting in such outcomes (Blair, 2007, 2013). However, research on psychopathy in both offender and non-offender samples has shown that the processing of various types of negative outcomes is disrupted in individuals with relatively high levels of psychopathic traits (von Borries et al., 2010; Brazil et al., 2017), ultimately affecting how they learn from social encounters (Blair, 2007, 2013; Brazil et al., 2011). People with elevated levels of psychopathy exhibit multiple impairments in moral decision-making (Driessen et al., 2021), including greater utilitarian thinking on sacrificial moral dilemmas, and a tendency to pursue personal advantage even when it causes pain to others in some way (Pletti et al., 2017). Usually, the prospect of pain triggers a psychological response in the form of an unpleasant negative emotional state (i.e., pain distress) (Puntillo et al., 2018), but this process could be disturbed in individuals with elevated levels of psychopathic traits. Indeed, it has been proposed that a diminished responsivity to others’ distress plays a key role in understanding poor moral decision-making in psychopathy (Blair, 1995, 2013). This notion has received support from studies linking psychopathy to reduced autonomic responding to the distress of others (House and Milligan, 1976), reduced recognition of faces signaling distress (Blair, 2007), and reduced empathic resonance of sadness, fear, and pain (Blair, 2013). Moreover, it has been proposed that the limited empathic resonance observed in individuals with elevated levels of psychopathy comes from a restriction in their own range of emotionality (Bird and Viding, 2014; Hoppenbrouwers et al., 2016). The assumption is that we understand others’ affective states to the extent to which we have access to information about our own affective states. Thus, our *own* range of affective experience functions as an “internal ruler”, and the boundaries of the internal ruler determine the extent to which we can estimate the intensity of what others may feel (Hoppenbrouwers et al., 2016).

Applied to the context of moral decision-making in psychopathy, a diminished capacity to estimate distress in others could be a consequence of a limited experience of distress at first hand when making choices that cause harm and pain. So, the painful consequences of one’s choices may trigger less distress in the individual as the presence of psychopathic traits increases, which makes it difficult to estimate how much pain distress others may experience. However, a direct application of the “self-to-other principle” for testing the role of pain distress during moral decision-making, and links with psychopathy and its facets, is lacking.

Therefore, this study investigated whether own pain distress mediates the relationship between psychopathic traits and beliefs concerning how much distress others may experience. We first examined whether an elevated psychopathy score would predict lower levels of own distress, which in turn should predict beliefs about the level of pain distress others experience. Given the multifaceted nature of psychopathy, we then unpacked psychopathy into four sets of psychopathic traits and hypothesized that their elevated levels would, too, predict lower levels of own distress. The relationship between psychopathy and estimates of pain distress in others was expected to be mediated by estimates of own pain distress. In addition, exploratory zero-order correlation analyses were conducted to examine how estimates of own and others’ pain distress, respectively, correlated with individual facets of psychopathy and the moral permissibility across different dilemmas.

## Materials and Methods

### Participants

A total of 192 participants were initially recruited at the university and *via* social networks. Of these, 18 were excluded due to missing data, resulting in a final sample consisting of 174 individuals (50.6% men) (Table 1). All participants provided written informed consent, and no financial compensation was given for their participation. The study was approved by the local ethics committee.

**TABLE 1:** Mean scores, standard deviations (SD), and Bayesian Pearson *r* correlations (95% CI between brackets) for the SRP-SF subscale scores, pain belief ratings, and moral permissibility scores.

	Mean (SD)	r (95% CI)					
		1	2	3	4	5	6
(1) SRP interpersonal	14.1 (5.02)	–	0.647* (0.547, 0.728)	0.583* (0.472, 0.676)	0.317* (0.171, 0.449)	−0.224* (−0.364, −0.072)	−0.131 (−0.278, 0.022)
(2) SRP affective	12.5 (3.99)	0.647* (0.547, 0.728)	–	0.656* (0.558, 0.736)	0.480* (0.353, 0.590)	−0.210* (−0.352, −0.059)	−0.059 (−0.211, 0.096)
(3) SRP lifestyle	15.2 (4.6)	0.583* (0.472, 0.676)	0.656* (0.558, 0.736)	–	0.500* (0.374, 0.606)	−0.278* (−0.413, −0.131)	−0.161* (−0.307, −0.007)
(4) SRP antisocial	10.4 (3.79)	0.317* (0.171, 0.449)	0.480* (0.353, 0.590)	0.500* (0.374, 0.606)	–	−0.152 (−0.299, 0.002)	−0.015 (−0.166, 0.138)
(5) Own pain distress	55.1 (16.8)	−0.224* (−0.364, −0.072)	−0.210* (−0.352, −0.059)	−0.278* (−0.413, −0.131)	−0.152 (−0.299, 0.002)	–	0.629* (0.525, 0.713)
(6) Others’ pain distress	60 (16.3)	−0.131 (−0.278, 0.022)	−0.059 (−0.211, 0.096)	−0.161* (−0.307, −0.007)	−0.015 (−0.166, 0.138)	**0**.629* (0.525, 0.713)	–
(7) Permissibility	49.5% (15.8)	−0.201* (−0.343, −0.050)	−0.207* (−0.350, −0.056)	−0.110 (−0.259, 0.043)	−0.130 (−0.277, 0.023)	0.070 (−0.081, 0.219)	0.008 (−0.143, 0.158)

*Indicate statistically significant correlations. The bolded values refer to significant correlations.

### Materials and Procedure

First, psychopathic traits were measured using the Dutch translation of the Self-Report Psychopathy scale-short form (SRP-SF; Gordts et al., 2017), which was administered *via* an online platform.^1^ The SRP-SF is a self-report questionnaire that is directly derived from the Psychopathy Checklist-Revised, which is the currently dominant instrument for the diagnosis of psychopathy (Hare, 2003). The SRP-SF consists of 29 questions that are rated on a 5-point Likert scale. The list yields scores on four facets that capture the interpersonal, affective, lifestyle and antisocial features of psychopathy, respectively, which can be summed to create a total score indexing the overall presence of psychopathic traits if desired (Neumann et al., 2012; Paulhus et al., 2016).

Next, participants were invited to participate in the experimental session. During this session, participants completed the Dutch version of the widely used moral dilemma task (Greene et al., 2001; Cima et al., 2010). In this task, participants were asked to read 21 scenarios that each described a moral dilemma, and they had to judge whether the dilemmas were morally permissible or not. Each scenario contained a dilemma that could lead to detrimental consequences for some of the individuals involved, making them the victims. For each dilemma, participants had to respond with “yes” or “no” to indicate whether they found the choice they had to make permissible (e.g., “Is it more permissible for you to flip the switch and kill one person to avoid killing the five workmen?”). As the main purpose of our study was to investigate the role of beliefs about pain-related distress in such moral decisions in relation to psychopathy, each participant was also asked to imagine that the dilemma in each scenario was real and to provide ratings on a scale ranging from 1 to 100 for each of 2 questions: (i) “How much psychological pain would *you* feel because of the choice you made?”, quantifying beliefs about own pain distress, and (ii) “How much psychological pain do you think *the victim(s)* would feel because of your choice?”, capturing the participant estimates of the pain distress experienced by others involved in the scenario. The scenarios were presented in pseudo-randomized order across participants.

### Statistical Analyses

First, general performance measures were computed. So, for each participant, an overall permissibility score was obtained by calculating the proportion of dilemmas deemed permissible. Average pain distress scores for both the self and others were calculated for each participant and compared using the Friedman non-parametric test given the non-normal distribution of the variables.

Next, we tested our main hypothesis by conducting a path analysis in Mplus v7.4 (Muthén and Muthén, 2017). We tested two different models: one in which the total SRP-SF score was used as a predictor of the average ratings of own pain distress, and one where the four facets of the SRP-SF were defined as the predictors of the own pain distress ratings. In both models, the own pain distress ratings predicted the average ratings of others’ distress. In both cases, a Bayesian estimator (PX1) was used to determine the regression weights and the corresponding 95% credibility intervals (CI), using Markov Chain Monte Carlo (MCMC) sampling with two Markov chains and 75,000 iterations. The first half of the iterations were considered burn-in trials to train the model and were excluded. Model fit was determined based on multiple Bayesian indexes: (i) The posterior predictive *p*-value (PPP value), which should approach the value 0.5, (ii) a posterior predictive check using χ^2^ testing, which indicates good model fit when the 95% CI interval of the χ^2^ test includes the value 0, and (iii) convergence of the MCMC chains based on the proportional scale reduction (PSR) factor, which should have a value <1.05 (Brazil et al., 2017; Muthén and Muthén, 2017). The significance of the regression weights within the model was determined based on the 95% CI, which should not contain the value of exactly 0. Both direct and indirect effects were tested.

Finally, Bayesian zero-order correlations were conducted to explore the correlations between the SRP facet scores, distress ratings and the permissibility scores. Significance was determined based on the 95% credibility interval (CI) for each correlation pair.

## Results

The mean SRP-SF score was 52.2 (SD = 14.04). The means and SDs for the individual facets can be found in Table 1. The distribution of SRP-SF scores was similar to that reported in prior studies (Riopka et al., 2015; Gordts et al., 2017; León-Mayer et al., 2019), with SRP-SF scores ranging from 29 to 109 (7 to 29 for the interpersonal, 7 to 25 for the affective, 7 to 27 for the lifestyle, and 7 to 36 for the antisocial).

On average, participants found the moral dilemmas to be permissible on 49.5% (SD = 16) of the trials. The average ratings of one’s own pain distress (mean = 55.1%, SD = 16.8) were lower than for the beliefs about distress experienced by others (mean = 60%, SD = 16.3) on the moral dilemmas [Friedman χ^2^(1) = 15.7, *p* < 0.001] (see also Table 1).

### Path Analysis

The model fit for the SRP-SF-total model was excellent (95% CI −10.772 to 10.661, PPP = 0.502). The results indicated that the total SRP-SF score was a significant negative predictor of the own pain distress ratings (β = −0.268, 95% CI −0.395 to −0.125). The indirect effect between the total SRP-SF score and ratings of others’ pain distress, mediated by the own pain distress ratings, was also significant (β = −0.166, 95% CI −0.256 to −0.076^2^).

**FIGURE 1:**
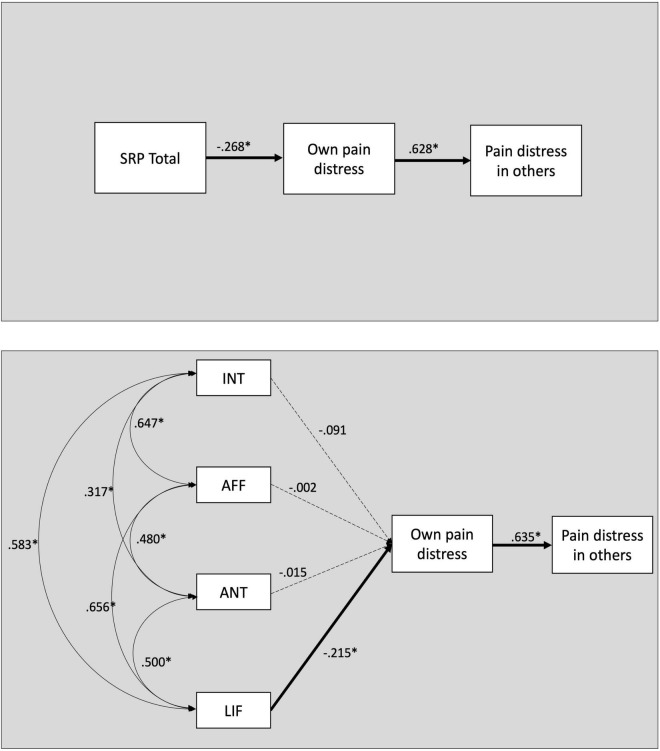
Bayesian path model with standardized regression weights. Significant paths are indicated with solid arrows. **Top panel:** model using psychopathy total scores as predictor. **Bottom panel:** models using the 4 facets of psychopathy as predictors. INT, Interpersonal facet; AFF, Affective facet; ANT, Antisocial facet; LIF, lifestyle facet.

The deconstructed 4-facet SRP-SF model also demonstrated an excellent fit (95% CI −20.233 to 20.534, PPP = 0.484). The results indicated that only the lifestyle facet was a significant unique predictor of own pain distress (β = −0.215, 95% CI −0.417 to −0.006). The interpersonal (β = −0.091, 95% CI −0.286 to 0.108), affective (β = −0.002, 95% CI −0.221 to 0.215) and antisocial (β = −0.015, 95% CI −0.187 to 0.159) facets were not statistically significant predictors of own distress (see also Figure 1). Additionally, ratings of own pain distress significantly predicted the estimates of pain distress in others (β = 0.635, 95% CI 0.537 to 0.717). The indirect effect between the lifestyle facet and others’ pain distress, mediated by own pain distress, was also statistically significant (β = −0.135, 95% CI −0.270 to −0.004).

### Correlations

The exploratory zero-order Bayesian correlations indicated that all the SRP-SF facet scores showed statistically significant negative correlations with the ratings for own pain distress, except for the antisocial facet (see Table 1). In contrast, only the negative correlation between the lifestyle facet and beliefs about others’ pain distress was statistically significant. The positive correlation between ratings for own and others’ pain distress, respectively, was also statistically significant. Finally, permissibility scores were significantly correlated with the interpersonal and the affective facet, with a negative effect size.

## Discussion

The main goal of the present study was to examine if own pain distress mediated the relationships between psychopathic traits and estimates of the level of pain distress that others may experience, in the context of moral decision-making. Partially in line with our predictions, all psychopathy facets apart from the antisocial facet correlated negatively with ratings of own distress. Lending support to the self-to-other principle, stronger own pain distress predicted higher estimates of the amount of pain distress experienced by others when making moral choices. While the total psychopathy score predicted lower levels of own pain distress, interestingly, in the more specific facet-based path model, only scores on the lifestyle facet uniquely predicted own pain distress. In turn, own pain distress mediated the indirect relationship between the lifestyle facet and the pain distress others may experience.

First, these results suggest that the self-to-other principle offers a useful framework for understanding other-regarding processes in relation to psychopathy. It may seem somewhat striking that the lifestyle facet, and not the interpersonal and affective components, was the only predictor of pain distress. One explanation can be found by considering the behavioral tendencies that constitute the lifestyle facet more closely. The lifestyle facet is linked to personality traits such as sensation-seeking and disinhibition, both of which are related to aberrant own distress experience in individuals with elevated levels of psychopathy (Brislin et al., 2016). One hypothesis is that higher sensation-seeking, as captured by the lifestyle facet, may correspond to a reduced capacity to make inferences about one’s own level of pain distress as a strategy to prioritize feelings of reward that follow from thrill-inducing activities. Individuals with elevated scores on the lifestyle facet could prefer to focus on the positive experiences that follow such activities (i.e., “feeling the rush”), while placing less emphasis on the aversive consequences, such as psychological distress, but perhaps also on feeling guilt, anxiety, shame (i.e., from being caught or from dealing with the social consequences, such as ostracizing or judgement). This hypothesis is in (partial) agreement with prior research showing, for example, that the impulsive-antisocial component of psychopathy is related to hypersensitivity to reward in non-offenders (Buckholtz et al., 2010), that the representation of the subjective value of reward is amplified in psychopathic offenders (Hosking et al., 2017), and that offenders with elevated levels of psychopathy adapt their behavior based on level of reward but not punishment (Blair et al., 2004). Thus, it seems plausible that individuals scoring higher on the lifestyle facet are systematically biased towards favoring positive outcomes with high hedonic value while downplaying the subjective value of aversive outcomes, including distress. If true, the bias could help explain findings linking psychopathy to reduced learning from painful outcomes (e.g., Hare, 1965; Birbaumer et al., 2005; Brazil et al., 2017). However, this claim should be treated with caution, as it still remains to be determined how the trade-off between subjective valuation of reward and pain affect learning and decision-making in psychopathy.

In addition to the theoretical model we tested with the path analyses, we also examined the simple correlations between psychopathy scores, the two measures of pain distress, and moral permissibility scores. As expected, own pain distress and beliefs about other’s pain distress were positively correlated, with a medium effect size. We did not find any statistically significant correlations between permissibility scores and the two measures of pain distress. These findings might appear counterintuitive at first, as experiencing less distress has been linked to making more utilitarian choices (Sarlo et al., 2014). However, the relationship between moral permissibility, pain distress, and behavior might be more complicated than that. Firstly, harm-centric approaches to cognition suggest that an action is judged as “morally wrong” when it leads to pain in others (Piazza et al., 2019), but alternative accounts point out that people often find harmful acts acceptable (Piazza and Sousa, 2016). So, whether a decision is morally permissible or not might be shaped by appraisals that go beyond just the perceived pain and distress it may cause (to the self or others). Some authors have suggested that strategies to reduce psychological distress might not necessarily be prosocial or what we consider “permissible” (Eisenberg et al., 2010). For example, seeing individuals in need might cause distress and one might choose to alleviate it by avoiding such individuals altogether, rather than helping them out. In any case, our results suggest that the permissibility of an action has no relationship with how much pain distress it elicits in the individual, or how much pain distress we believe others would be in as a result of the decision.

Our correlational findings also indicate that the increased presence of the lifestyle component was the only feature of psychopathy significantly correlated with lower estimates of pain distress in others. We also found that own pain distress diminishes as the interpersonal, affective and lifestyle components of psychopathy become more prominent. As distress captures negative affective states (Sarlo et al., 2014; Pletti et al., 2016), our results agree with prior research showing alterations in the processing of affective information as the presence of psychopathic traits becomes stronger (Marsh and Blair, 2008; Dawel et al., 2012). These findings also converge with prior findings indicating an altered experience of physical pain as psychopathic traits become more prominent, including heightened pain tolerance and diminished sensitivity for physical pain in non-offenders (e.g., Miller et al., 2014; Brislin et al., 2016). In offenders, there is evidence for reduced spontaneous vicarious representations in response to videos depicting painful interactions (Meffert et al., 2013) and reduced brain activity in emotion-relevant regions during passive viewing of negatively salient content (Shane and Groat, 2018).

Finally, statistically significant negative correlations were found for the interpersonal and affective facet with moral permissibility. These results are surprising as they suggest that with the increase in interpersonal and affective traits, the number of moral dilemmas deemed permissible decreases. This goes against findings of increased moral permissibility in offenders with high levels of psychopathy (Young et al., 2012). However, the absence of relationship between the interpersonal and affective facets with permissibility judgements could be seen as another indication that participants employed decision-making strategies in which engaging in a harmful act was not necessarily considered to be unacceptable or impermissible (Piazza and Sousa, 2016).

Despite the advances offered by our results, it still remains important to consider that the present study concerned self-reported pain distress. While self-report methods are generally valid tools for assessing the experience of distress and pain (Robinson et al., 2013), coupling these with neurobiological (e.g., brain activation) and psychophysiological measures of distress processing may provide more robust information. Another consideration is that it remains to be established whether our results remain the same when other correlates of pain are assessed (e.g., imagined pain, sensory pain, pain threshold), in addition to pain-related distress. Pain is a complex and multifaceted construct, with distinct correlates across multiple domains (e.g., sensory, cognitive, and affective) (Wiech, 2016; Becker et al., 2018). This complexity, along with the observation that the experience of pain is highly subjective, makes is particularly challenging to reliably measure the various aspects of pain. The challenge is nicely illustrated by findings showing that psychopathic traits exhibit different patterns of associations with physical pain, depending on the type of pain measurements used (Miller et al., 2014). One solution could be to collect and fuse measurements of pain across domains in each study in the future. Finally, while our participants displayed a good range of psychopathy scores (albeit sub-clinical), it is not necessarily clear how the findings from a community sample will translate to clinical or forensic populations. Therefore, it might be worthwhile to replicate the experiment in a clinical setting.

In conclusion, our findings provide support to the “internal ruler” hypothesis, suggesting we perceive distress in others to the extent that we, ourselves, are prone to experiencing distress. Moreover, the present study provides the first evidence that an elevation in scores on the lifestyle facet of psychopathy co-occurs with a tendency to experience less pain distress when making moral choices, as well as judging the pain distress that other may possibly experience as being less severe. This hints toward a restricted range of aversive experience, both when others and the self are considered. It still remains to be shown how exactly this limited range might translate into poor moral choices. The latter will likely require experimental novel approaches that can be combined with the use of computational models that can dissociate the underlying latent processes (Brazil et al., 2017; Seymour, 2019; Driessen et al., 2021).

## Data Availability Statement

The raw data supporting the conclusions of this article will be made available by the authors, without undue reservation.

## Ethics Statement

The studies involving human participants were reviewed and approved by the Ethics Committee of the Faculty of Social Sciences (ECSS), Radboud University, the Netherlands. The patients/participants provided their written informed consent to participate in this study.

## Author Contributions

IB and JO conceived of the presented idea and developed the theoretical framework. IB carried out the experiment and performed the related analyses. DA wrote the manuscript with support from IB and JO. All authors contributed to the final version of the manuscript.

## Conflict of Interest

The authors declare that the research was conducted in the absence of any commercial or financial relationships that could be construed as a potential conflict of interest.

## Publisher’s Note

All claims expressed in this article are solely those of the authors and do not necessarily represent those of their affiliated organizations, or those of the publisher, the editors and the reviewers. Any product that may be evaluated in this article, or claim that may be made by its manufacturer, is not guaranteed or endorsed by the publisher.

## Footnotes

1
www.thesistools.com

2 Because the lower bounds of some of the 95% CI were very close to zero for some of the regressors, we re-analyzed the model using 200,000 iterations instead of 75,000 to establish the stability of the initial results. The results remained unchanged, further supporting the validity of the results. As a second additional check, we also ran the analyses using a robust maximum likelihood estimator, which yielded identical results.
